# Noninvasive characterization of pancreatic tumor mouse models using magnetic resonance imaging

**DOI:** 10.1002/cam4.1062

**Published:** 2017-04-07

**Authors:** Navid Farr, Yak‐Nam Wang, Samantha D'Andrea, Kayla M Gravelle, Joo Ha Hwang, Donghoon Lee

**Affiliations:** ^1^ Department of BioengineeringUniversity of WashingtonSeattleWashington; ^2^ Applied Physics LaboratoryUniversity of WashingtonSeattleWashington; ^3^ Department of MedicineUniversity of WashingtonSeattleWashington; ^4^ Department of RadiologyUniversity of WashingtonSeattleWashington

**Keywords:** Apparent diffusion coefficient, fibrosis, KPC mouse model, magnetic resonance imaging, magnetization transfer ratio, pancreatic ductal adenocarcinoma

## Abstract

The preclinical models of pancreatic adenocarcinoma provide an alternative means for determining the mechanisms of malignancy and possibilities for treatments, thus representing a resource of immense potential for cancer treatment in medicine. To evaluate different tumor models, quantifiable magnetic resonance imaging (MRI) techniques can play a significant role in identifying valuable in vivo biomarkers of tumor characteristics. We characterized three models of pancreatic cancer with multiparametric MRI techniques. Tumor stromal density of each tumor was measured using diffusion‐weighted imaging and magnetization transfer (MT‐MRI). Histologic measurement showed a similar trend with tumor fibrosis levels. Results indicated that MRI measurements can serve as a valuable tool in identifying and evaluating tumor characteristics.

## Introduction

Pancreatic ductal adenocarcinoma (PDA) is among the most lethal cancer worldwide. In addition, PDA is among the types of cancer in which death rates have risen in the 21st century 1. The lethal nature of the disease is apparent in the 5‐year survival rate, of <6%, for those diagnosed with PDA, while the median survival time is only 4–6 months 1. The current standard of care for metastatic pancreatic cancer is gemcitabine. Despite this, it has only been shown to improve the quality of life in a minority of patients while only prolonging survival by several weeks 2, 3. Oncology drug development relies heavily on tumor‐bearing mouse models for testing efficacy of novel therapeutic agents. Therefore, it is essential to validate and characterize mouse models of PDA, which have properties similar to those of human PDA, before testing various therapeutic modalities. Xenograft mouse models of PDA respond to numerous chemotherapeutic agents, including gemcitabine 4, 5, 6, 7, 8, but these agents have not had similar effects in human PDA 9, indicating the poor predictive utility of these models.

As an experimental model, tumors have been implanted subcutaneously primarily in mice with fast growth rates and ease of access for interventional treatment. However, the disease progression in these animal models does not parallel the human disease because the mouse tumors are often surrounded by a pseudocapsule and rarely metastasize 10. Furthermore, no adjacent major anatomic structures (such as major vessels or gastrointestinal organs) are invaded by the tumor in these mice 11. An orthotopic pancreatic tumor model was established by Tan and Chu by inoculating human pancreatic tumor ASPC‐1 cells in the pancreas 12. Orthotopic pancreas tumors have provided an experimental system that confirms the tumorigenicity and metastatic incidence of many human tumors in vivo. Compared to human pancreatic cancer, however, the orthotopic xenografts are not poorly perfused 13. An alternative to implanted cells and tumors is the genetically engineered mouse model of PDA that more closely resembles the clinical features, histopathology, and molecular progression of human pancreas cancer from inception to invasion. *Kras* ^LSL‐G12D/+^, *Trp53* ^LSL‐R172H/+^, and *Pdx‐1*‐*Cre* (KPC) mice conditionally express physiologic levels of oncogene and tumor suppressor gene mutations in pancreatic progenitor cells, resulting in the development and spontaneous progression of preinvasive ductal lesions to invasive and metastatic carcinomas 14, 15.

A common indication of PDA is tumor desmoplasia, characterized by dense fibrotic connective tissue that penetrates and envelopes the neoplasm. Different studies have shown that this extracellular matrix is not a passive scaffold but that it actively facilitates disease progression 16, metastasis 9, and drug resistance 13. The previously mentioned experimental mouse models, subcutaneous‐, orthotopic transplant and transgenic, have different tumor fibrosis levels which leads to diverse treatment outcomes.

There is a dire necessity for noninvasive techniques to monitor and characterize the tumors in different models to properly design drug delivery studies. Characterizing the tumor tissue can be used to predict the response to chemotherapy 17. In targeted drug delivery applications, evaluating tumor characteristics lead to better targeting of the tumor by avoiding liquid cystic areas and the necrotic cores of the tumor. The identification of prognostic factors before treatment would be helpful in selecting the subgroups of patients for which chemotherapy improves survival and in determining efficient treatment strategies with reference to expected survival 18. Magnetic resonance imaging (MRI) is excellent in the delineation of small pancreatic tumors due to its superior soft tissue contrast. Additionally, MRI is more effective at detecting cystic masses or pancreatitis than computer‐assisted tomography (CT).

Anatomic T2‐weighted (T2W) and T1‐weighted (T1W) MRI has been widely used to identify pancreatic malignancies 19, 20, 21. He et al. used serial T2‐weighted MR images to construct the tumor growth curves and measure the tumor therapeutic response 22. In vivo MRI can be sensitized to local characteristics of water diffusion by utilizing the Brownian characteristic of water molecules 23, 24, 25. Hence, diffusion‐weighted MRI has served as a noninvasive biomarker to differentiate healthy pancreas from pancreatic cancer 26. Moreover, multiple studies in cartilage degeneration 27 and regeneration 28, liver fibrosis 26, and fibrosis levels in PDA 29 have each demonstrated that magnetization transfer (MT) contrast can be highly sensitive to tissue collagen concentration which is the primary component of desmoplasia 30.

The purpose of this study was to characterize the different properties of PDA tumors in three different mouse models (genetic, subcutaneous xenograft, and orthotopic xenograft). Providing information on the tumor characteristics could help in identifying the aggressiveness of the disease and potentially help in the determination of the most efficient treatment. In this study we have evaluated the T1‐ and T2‐weighted MRI to accurately identify and measure the size of pancreatic tumor. Additionally, we used quantifiable MR techniques such as quantitative T2 measurement, diffusion‐weighted imaging (DWI), apparent diffusion coefficient (ADC), and MT MRI to characterize the tumor in three mouse models.

## Materials and Methods

### Animal and tumor model

All experimental procedures were approved by the Institutional Animal Care and Use Committee (IACUC) at the University of Washington. In addition to the KPC mouse model, which spontaneously develops pancreatic ductal adenocarcinoma, orthotopic and subcutaneous xenograft mouse models were also used for this study.

Methods to produce orthotopic tumors were based on those developed by Tseng et al. 31. Eight‐ to 10‐week‐old immunocompetent mixed *129/SvJae/C57Bl/6* mice were anesthetized and prepared for sterile surgery. A 2‐cm incision was made along the left flank to expose the tail of the pancreas. One million cells derived from liver metastases of KPC mice in 40 *μ*L Matrigel were injected directly into the pancreatic parenchyma, and the incision was then closed with sutures. The animals were recovered and tumor growth was monitored by ultrasound and palpation. For the subcutaneous mouse model, the same procedures were followed with the exception of injecting the tumor cells subcutaneously into the flank, close to hind limb. Tumor development was monitored visually and with palpation. Prior to MRI scans, all tumors were monitored by measuring the average tumor diameter, in perpendicular axis of the tumor, by ultrasound or calipers. Eight mice in each tumor group (orthotopic, subcutaneous xenograft, or transgenic) were scanned using MRI.

### MRI procedures

For in vivo imagining, high‐resolution MRI acquisitions were performed on a 14T Avance 600 MHz/89 mm wide‐bore vertical MR spectrometer with a microimaging accessory (Bruker BioSpin corp., Bellerica, MA) in combination with ^1^H radiofrequency (RF) birdcage coil (inner diameter of 25 mm) and the coil holder. The high‐field MRI has the benefits of higher signal‐to‐noise ratio, contrast‐to‐noise ratio, and spectral resolution for certain application. In most cases, these benefits facilitate higher spatial and temporal resolution.

Mice were imaged when tumors reached a size between 500 mm^3^ and 1000 mm^3^ in volume. As previously described 32, once anesthetized and eye lubricant was applied, the mouse was placed into a radiofrequency coil and secured to a cradle created specifically for the MRI system. The coil was then inserted vertically into a scanner heated to maintain thermoneutrality (32°C). The coil was equipped with an adjustable anesthetic flow and vacuum system to maintain sedation throughout the experiment. Total scan time was 30–45 min, during which respiration was monitored through a respiration sensor under the abdomen (SA Instruments Inc., Stony Brook, NY) and anesthesia titrated to ensure appropriate sedation. Following the imaging paradigm (described below), mice were removed from the coil and allowed to recover in their respective cages.

### MRI protocol

The high‐resolution MRI protocol includes scout imaging (gradient echo; repetition time (TR)/echo time (TE) = 30/1.3 msec), planning for image planes (multislice rapid acquisition with refocused echoes (RARE): TR/TE = 668/4.5 msec), high‐resolution two‐dimensional imaging with 30 thin slices (200 micron thick) (multislice RARE: TR/TE = 4000/6 msec) for tumor volume evaluation, multislice multiecho imaging (TR/TE = 4000/6–100 msec, 16 echoes with 6.3 msec spacing) for transverse relaxation time T2 measurements, magnetization transfer imaging (gradient echo; TR/TE = 939/5 msec, flip angle = 30°), and diffusion imaging with three *b*‐values of 0, 500, and 1000 sec/mm^2^ sequence (TR/TE = 5000/27.5 msec).

The quantitative T2 measurements utilized spin‐echo sequences to generate T2 maps; T2 maps were generated using a multislice multiecho sequence (TR/TE = 4 sec/6–100 msec, 16 echoes) with a suppressed fat signal (Gaussian pulse, pulse length = 1.3 msec, bandwidth = 2100.5 Hz) at 14T. The T2 fit maps were generated using SI = *Ae* ^−TE/T2^ function, where SI is the signal intensity and *A* is the amplitude. Turbo spin‐echo T2‐weighted contiguous images were acquired in the coronal plane, with fields of view (FOV) of 40 × 40 mm. T2‐weighted images were used to visually inspect the tumor for apparent signs of necrosis and cystic regions and to measure comparable regions of interest in tumor and surrounding organs.

Diffusion‐weighted imaging was performed in a coronal plane using a spin‐echo imaging sequence (TR/TE = 5000/27.5 msec) with three diffusion gradients (*b* = 0, 500, and 1000 sec/mm^2^). The diffusion gradients were oriented along the direction of the slice section gradient. The diffusion MRI was conducted using the spin‐echo sequence as described by Le Bihan et al. 33, 34. ADC maps were generated by carrying out diffusion‐weighted imaging utilizing the *b*‐values using signal intensity *S* = *S*_0_ *e* ^−b*ADC^ function, where *S*_0_ is the signal intensity with no diffusion gradient (*b* = 0). In order to have a baseline and distinct value from the tumor, the ADC values of the tumor were compared with the values of the liver, spleen, and kidney.

Magnetization transfer suppression ratios or MT ratios (MTR) were calculated by the method described by Li et al. 29. Briefly voxel‐wise MTR maps were calculated as follows: 100 × (1−*S*/*S*_0_), where S represents the signal intensity for the image acquired following application of the MT pulse and *S*_0_ is the signal intensity image acquired without MT saturation. We utilized a gradient echo sequence (TR/TE = 939/5 msec, flip angle =30°) with an offset frequency of 7000 Hz and a saturation block pulse (50 msec width and 10 *μ*T amplitude).

During the scans the mice were monitored to minimize motion effects in the imaging process via image gating. This was accomplished by using the MR‐compatible small animal monitoring and gating system (SA Instruments Inc., Stony Brook, NY). The system was setup to monitor the respiration and ECG gating. After initial studies demonstrated that ECG gating was superfluous, only respiration gating was used to remove motion artifacts by triggering during the MR acquisition.

### MRI data analysis

All raw MR images were processed using Image‐J software (Rasband, W.S., ImageJ, U. S. National Institutes of Health, Bethesda, ML, http://imagej.nih.gov/ij/, 1997–2012) 35, to measure mean values of the different tumors and other areas such as the muscle and spleen. Regions of interest (ROI) were drawn to circumscribe the entire tumor. 3D reconstructions of the tumors were completed using Amira (Visualization Sciences Group, Burlington, MA).

### Histology

Microscopic morphologic analysis was performed using standard tissue histology methods. Tumors were excised, embedded in optimum cutting temperature medium, and 5 *μ*m sections were taken (CM1950, Leica, Bannockburn, IL). Serial sections of two locations in each tumor taken from top and middle layer, stained with Hematoxylin and eosin (H&E) or Masson's trichrome using standard protocols. The H&E stain was used for gross histological assessment of necrosis. Masson's trichrome was used to visualize the stromal matrix. The sections were examined using a Nikon H550L light microscope (Nikon, Melville, NY) and images taken from the entire slides.

Masson's trichrome images were analyzed using Matlab software (MathWorks, Natick, MA) with fibrotic tissue areas being quantified within each slide accounting for the different color staining. Fibrotic stroma was depicted as blue‐stained bands of collagen and the tumor cells were identified by the stained nucleus. The percentage of fibrotic tissue was defined as the ratio *A*_fibrosis_/*A*_total_ × 100. *A*_total_ is the total tumor tissue area and *A*_fibrosis_ is the total fibrotic area. These measurements were repeated for each animal. The percentages were compared with the MT ratio for each group.

### Statistical analysis

In order to effectively resolve differences between different tumor models and their properties, eight animals in each of the three groups were measured.

All quantitative MRI data and histologic data were expressed as mean ± SD. One‐way analysis of variance was used to compare MTR and histological measurements. Post hoc analysis was performed using the Tukey method. Pearson correlation coefficients were calculated to assess the relationship between MTR measurements and corresponding histologic fibrosis measurement. Statistical analyses were performed with Prism software (Prism 6, GraphPad Software, Inc., La Jolla, CA) and Stata software (Stata 11, Stata‐Corp, College Station, TX).

## Results

**Figure 1:**
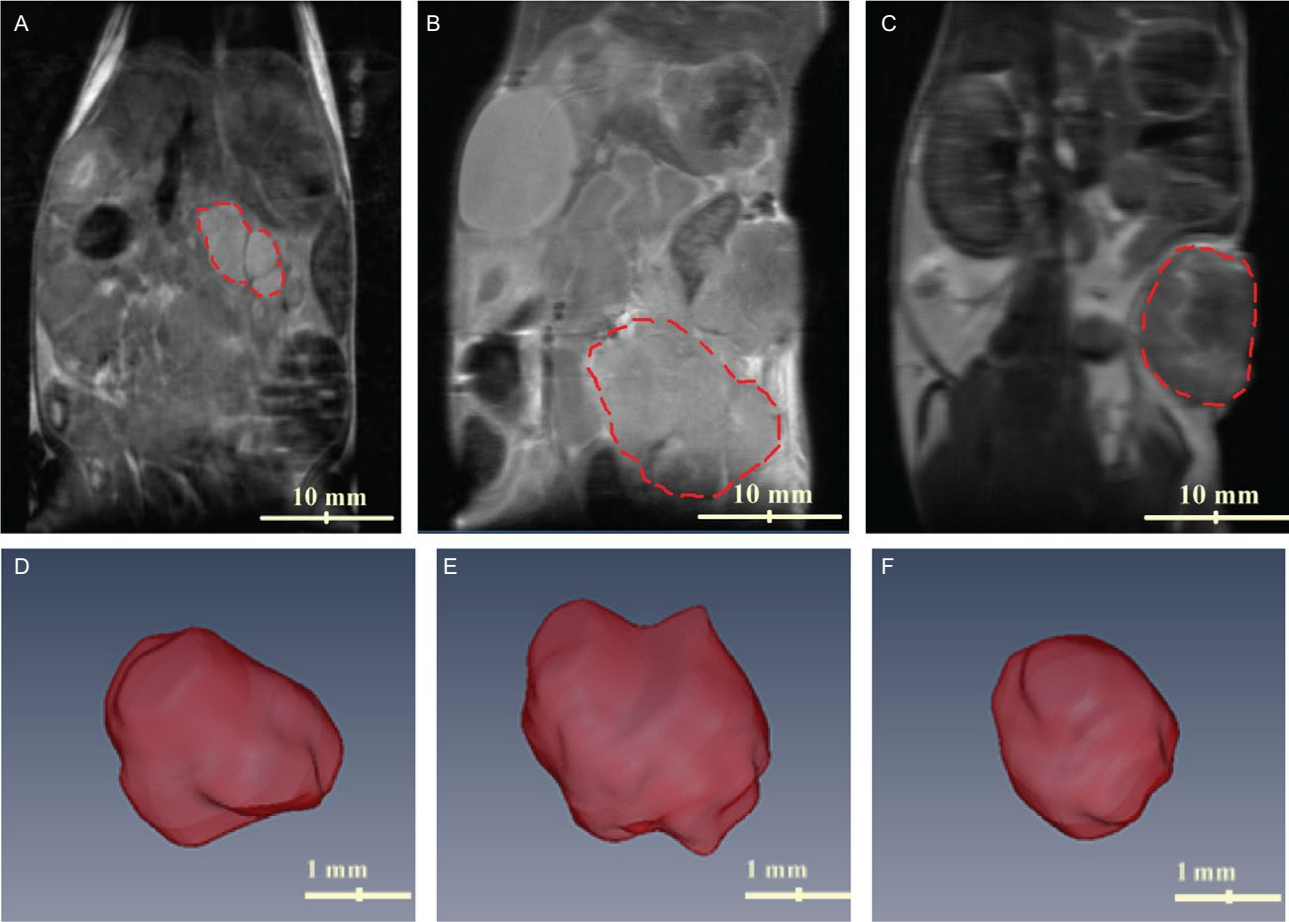
T2‐weighted images of three different mouse models in coronal view. (A) KPC tumor, (B) orthotopic tumor, and (C) subcutaneous tumor mice. Red dashed line identifies the tumor growing on the flank (C), tail of pancreas (B), and pancreas (A). Representative 3D volumetric image of the tumor grown in each model can be used to calculate the volume of the tumor. (D) KPC, (E) orthotopic, and (F) subcutaneous tumors.

Figure 1 shows the coronal view T2W images of three different tumor models. The red dashed line indicates the tumor and surrounding organs are visible in the plane. By reducing the slice thickness and acquiring more slices, 3D reconstruction of each tumor was possible. The 3D image of each tumor is shown in Figure 1 and can be used to calculate the volume of each tumor.

**Figure 2:**
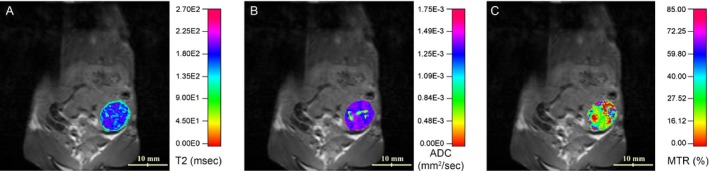
T2 relaxation time (ms) (A), ADC (mm^2^/sec) (B), and MTR (%) (C) of the tumor overlaid onto a T2‐weighted image showing heterogeneous distribution of the three quantitative MR parameters in a PDA tumor.

**Figure 3:**
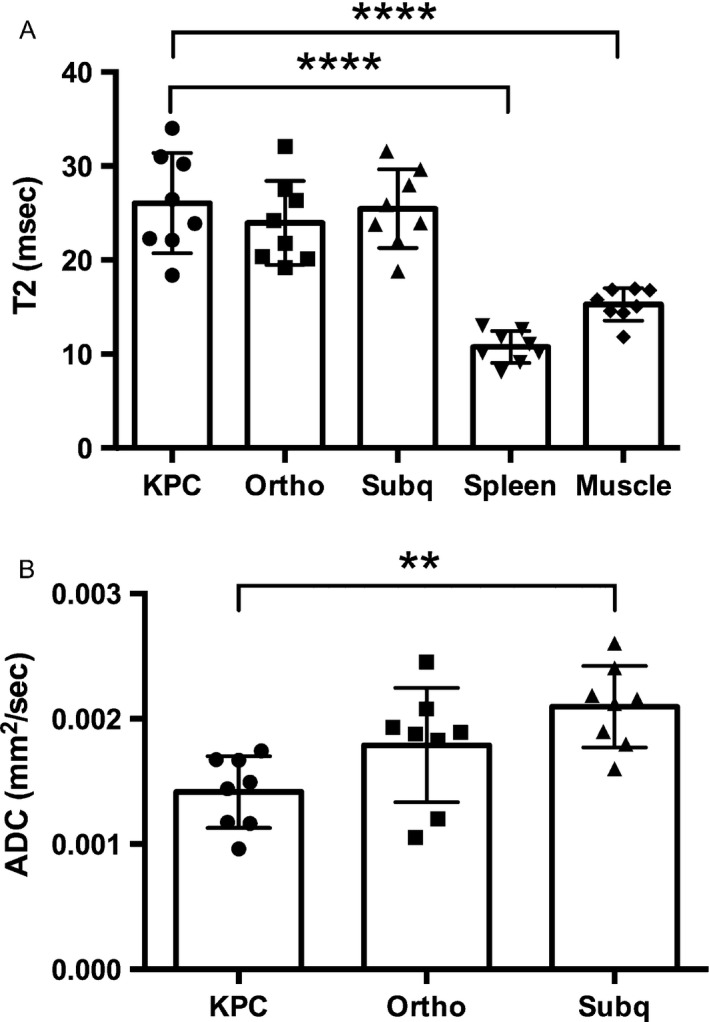
(A) T2 relaxation times measured for the three different mouse models and two organs (viz*.,* spleen and muscle) in all animals (*n *= 8 for each tumor model). (B) ADC values measured for the three different mouse models of KPC, orthotopic, and subcutaneous tumors (*n *= 8 for each tumor model). ** *P* < 0.01 and **** *P* < 0.0001.

Using fully automated acquisitions of T2 series, followed by a semiautomatic image analysis, a T2 map of the tumor was generated and overlaid onto the T2W image. A representative example is shown in Figure 2. The T2 relaxation time of the tumor in each model is shown in Figure 3A. There is no statistical difference among T2 relaxation times of tumor tissues in the three models; however, there is a difference in T2 relaxation times between tumor tissue and other organs (viz. spleen and muscle). In addition to the T2 map, the other quantitative ADC and MTR maps were generated and overlaid onto the T2W image as shown in Figure 2. Signal‐to‐noise ratio (SNR) values on the region of interest were estimated 9.1, 3.9, and 5.3 for raw images acquired with the longest TE (100 msec), *b* = 1000 sec/mm^2^, and MT saturation for T2, ADC, and MTR maps, respectively.

KPC mouse tumors were shown to have the lowest average ADC value (0.0014 mm^2^/sec) while the subcutaneous mouse tumors were shown to have the highest average ADC value (0.0021 mm^2^/sec) (Fig. 3B).

**Figure 4:**
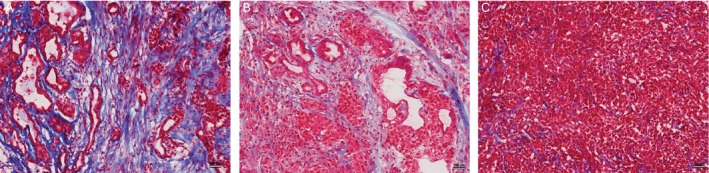
Masson‐trichrome‐staining histology sections from tumors grown in each mouse model. (A) KPC, (B) orthotopic tumor, and (C) subcutaneous tumor mice. Fibrotic stroma is depicted as blue‐stained bands of collagen. Each image shows the overall distribution of fibrotic tissue within these central tumor slices. Scale bar = 100 *μ*m.

The H&E stained sections were used for qualitative assessment of the tissue. Representative Masson's trichrome stained sections for KPC, orthotopic, and subcutaneous mice are shown in Figure 4. The KPC mice consistently demonstrated significantly higher levels of collagen tissue deposition; the percentages of fibrotic tissue area measure for these specific examples were 7.8, 5.21, and 1.4 for KPC, orthotopic, and subcutaneous tumors, respectively. These data were compared with the MTR measurements for each group of animals.

**Figure 5:**
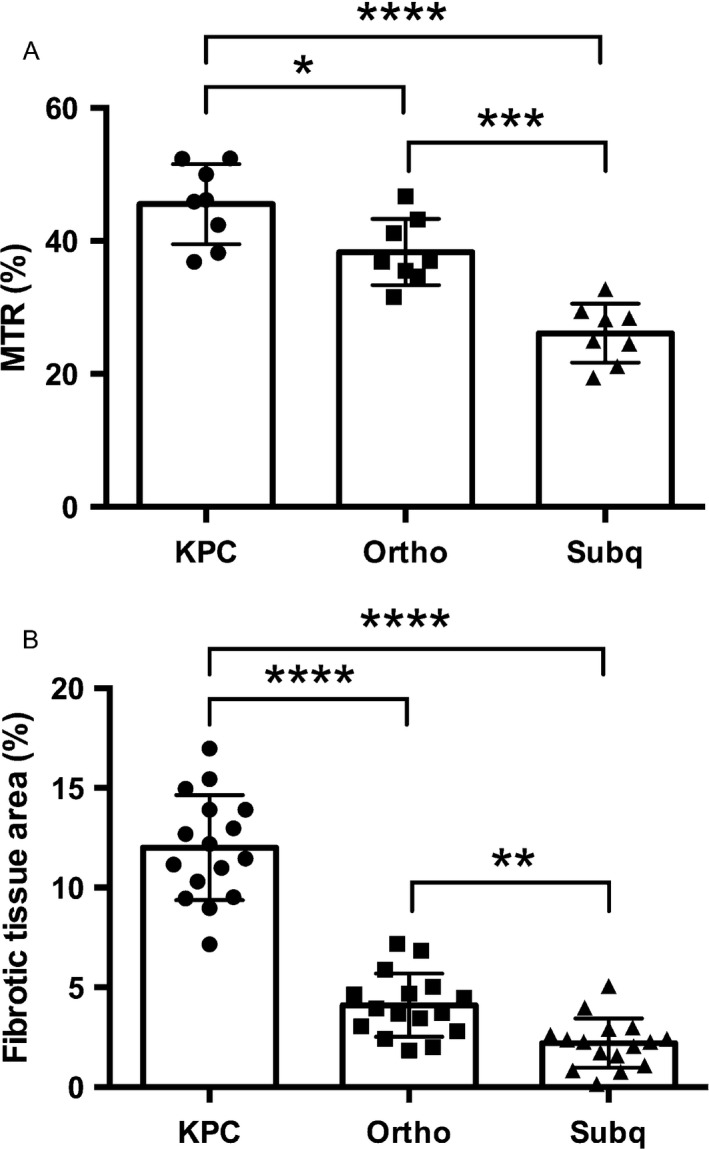
MTR measurements (A) and histologic fibrotic tissue area measurements (B) for each of the three mouse models. There is a correlation between MT ratio and the amount of stroma in the tumor (*r *= 0.88). * *P* < 0.05, ** *P* < 0.01, *** *P* < 0.001 and **** *P* < 0.0001.

**Figure 6:**
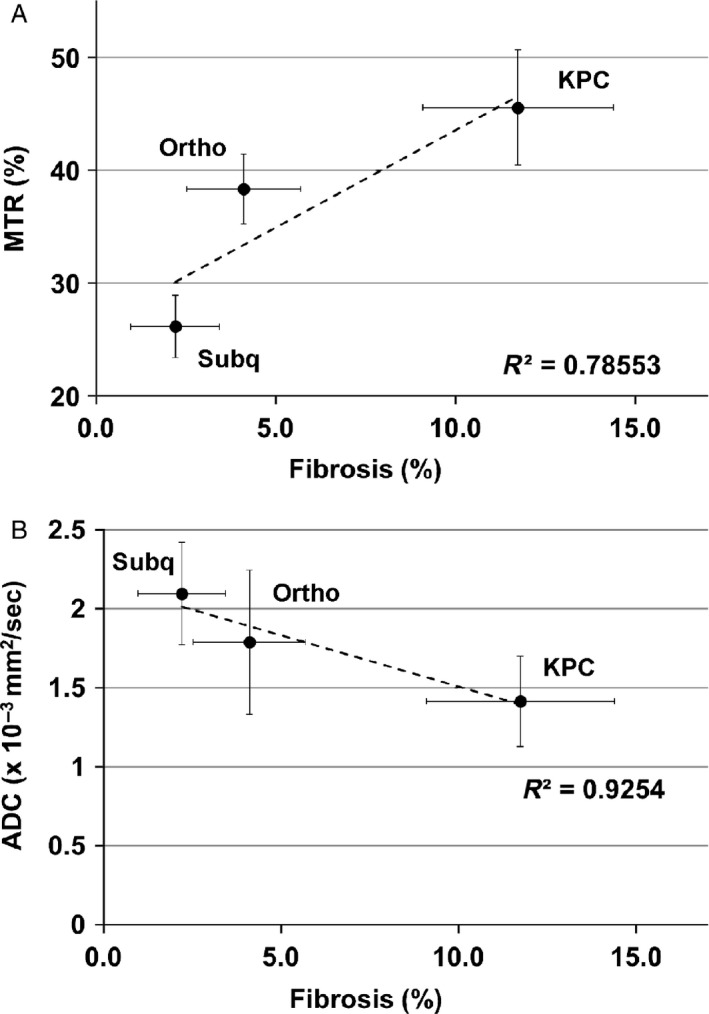
Correlation between MTR (A) (or ADC (B)) values and degree of fibrosis measured for the subcutaneous (Subq), orthotopic (Ortho), and KPC mouse models.

A summary of both MTR measurements and histologic measurements of fibrotic tissue area for each tumor type is shown in Figure 5. MTR measurements from the tumors in KPC mice (45.4 ± 5.1%) were higher than MTR measurements in orthotopic mice (38.4 ± 3%) and tumors in the subcutaneous mice (26.3 ± 2.8%). The comparison of MTR and histologic data indicates that there is a correlation (*r* = 0.89) between MT ratio and the amount of stroma in each tissue as shown in Figure 6. In addition, ADC values of tumors for the three mouse models were compared to the degree of fibrosis indicating a good correlation (*r* = −0.96) (see Fig. 6).

## Discussion

Specific characteristics of the tumor, such as desmoplasia and hypovascularity, play a significant role in the pathogenesis and drug‐resistance of PDA. Noninvasive methods used to monitor the collagen deposition associated with PDA desmoplasia may play an important role for understanding disease progression, predicting response to chemotherapy, or detecting response to therapies that target the tumor stroma. In this study, in vivo multiparametric MRI measurements were performed in three mouse models of PDA. These resulting MR measurements were significantly different among the established mouse models of pancreatic tumor, and corresponding histology measurements demonstrated significantly different collagen levels for each tumor type. These results affirmed prior studies which demonstrated that MT‐MRI measurements could be sensitive to tissue collagen content 29.

MTR values were correlated with histological slides of known desmoplasia status. The correlation between MTR and histology data suggests that MRI can directly predict desmoplasia level. The data confirm the previous reports that the transgenic KPC model has the highest degree of fibrosis in stroma compared to other types of tumor models evaluated 13. This MRI technique can be used to study the heterogeneous nature of tumor fibrosis and be used for PDA diagnosis. Future studies may be valuable to investigate the intratumoral heterogeneity of collagen deposition.

The result of this study reveals a significant difference between the ADC values of the tumor tissue in KPC, orthotopic, and the subcutaneous models. The ADC value in the DWI determines the motion of water protons in the measured environment. Lower ADC values may be explained, in part, by the accumulation of fibrotic tissue, which is the most characteristic feature of PDA, and might be associated with restricted motion of the water protons 26. There is also evidence to suggest that increased tumor cellularity causes restricted water diffusion on DWI, resulting in lower ADC values 36. Accordingly, our results reveal that the low ADC values of the tumor in KPC mouse might be associated with increased necrosis features of the tumor in comparison with the other tumor models 18. Diffusion MRI studies have been conducted for pancreatic cancer patients showing reduced ADC values (1.4–1.5 × 10^−3^ mm^2^/sec) compared to those for normal pancreas (1.8–2.1 × 10^−3^ mm^2^/sec) 26, 36, 37. Our ADC values measured for PDA in KPC mice were in the similar range (1.4 ± 0.3 × 10^−3^ mm^2^/sec) to that of human patients with pancreatic cancer. T2 measurements are helpful in detecting pathological tissues due to their long T2 relaxation time (e.g., degenerative changes in cartilage, muscular atrophy, and high signal intensity in tumors). The significant difference in the T2 relaxation time between the tumors in all models and the surrounding organs suggests this method is valuable for identifying and detecting the cancerous tissues.

In conclusion, our study on three mouse tumor models demonstrated that different MRI techniques can be used to characterize and identify the pancreatic tumor. MT MRI and DWI may be helpful as complementary imaging method to identify and characterize the tumor. Characterization of each tumor model reveals special feature of each tumor that enables research groups to choose the right model of different studies. For instance, the similarities in the barriers of drug delivery between KPC mouse model and PDA in humans make it a perfect candidate for development of new therapeutic agents and drug delivery studies. Finally, various MR parameters may provide useful information that will help in the understanding of pancreatic cancer biology and prove useful for the preclinical evaluation of new therapeutic agents or novel methods of treatment by enhancing the treatment planning and evaluation of treatment success and choosing the right mouse model for every preclinical study.

## Conflict of Interest

None declared.
